# Novel virus, novel response: Local discretion and responses to COVID‐19 in Hebei Province, China

**DOI:** 10.1002/app5.342

**Published:** 2022-01-10

**Authors:** Hong Gao, Adam Tyson, Guangxin Cheng

**Affiliations:** ^1^ School of Political Science and Public Administration China University of Political Science and Law Beijing China; ^2^ School of Politics and International Studies University of Leeds Leeds UK

**Keywords:** central‐local relations, China, COVID‐19, decentralisation, health policy

## Abstract

The Chinese Communist Party is consolidating one party rule under the leadership of Xi Jinping. Beijing seeks to rule by central mandate while limiting local autonomy. The central government response to the COVID‐19 public health emergency reinforces this view. In January 2020 Beijing established the Central Epidemic Response Leading Group to mobilise a comprehensive nationwide policy effort to contain the virus. The exceptional nature of the COVID‐19 national emergency allows the central government to project power over local authorities and leverage over citizens, but we argue that this is a short‐term phenomenon because local disease control initiatives remain important, with local authorities adapting national policies to meet constituent needs. There are degrees of policy discretion and divergence at the subnational level that enable context‐specific responses to the virus within China’s strict bureaucratic hierarchy. Primary data derives from interviews and observations in Nancun village, Hebei Province, conducted from January to April 2020. Evidence from Nancun explains how local authorities interpret the edicts and mandates of the central government.

## 1 INTRODUCTION

On 31 December 2019 the World Health Organization Country Office in China received the first reports of an unknown pneumonia outbreak in Wuhan Jinyintan Hospital. By mid‐January 2020 genomic sequencing identified the zoonotic novel coronavirus now known as COVID‐19, and asymptomatic carriers had already journeyed home from Wuhan to celebrate the Lunar New Year (Khanna & Honavar, 2020). Some five million people left Wuhan before travel bans were imposed (Chen et al., 2020). As China declared an epidemic and went into lockdown the virus began its long march, quickly becoming a global pandemic. There are serious concerns about China’s handling of the outbreak (Green, 2020), though the World Health Organization joint mission on COVID‐19 report shows the powerful scope of the government’s daily monitoring capacity through the National Reporting System and National Infectious Disease Information System (World Health Organization, 2020).

Under the leadership of Xi Jinping, who came to power in 2012, the Chinese Communist Party (CCP), in power since 1949, has further consolidated one party rule. Beijing is recentralising political authority, tightening its grip on key bureaucracies, and pushing broad national policy initiatives (Jaros & Tan, 2020, p. 79). In the context of the exceptional COVID‐19 national emergency response that is used by the central government to project its power over local authorities in the short term, this article asks to what extent local responses are decentralised by identifying forms of policy discretion and divergence at village level in Hebei Province. One peculiarity of the Chinese state is that despite its huge size (being continental in scale) and population, China has a centralised unitary system rather than a federalist system (Chung, 2016, p. 2). Even with persistent dynamic centrifugal forces challenging the centre, the functional and hierarchical aspects of central‐local relations show that China’s central state remains resilient. There is a complex balance between central government control and local‐level discretion.

In a move to consolidate central government rule, President Xi is restricting discretionary powers from local governments and intensifying monitoring and sanctioning practices (Kostka & Nahm, 2017). In policy terms the level of permissible local discretion depends largely on the scope, nature and urgency of policy (Chung, 2016, p. 90). When Chung’s (2016, p. 91) three dimensions of policy type are applied to ‘extreme contingencies’ such as the 2002–2003 Severe Acute Respiratory Syndrome (SARS)^1^ emergency, there is limited space for local strategic manoeuvring and discretion. As an ‘encompassing‐governance‐urgent’ policy with material, bureaucratic and ideological dimensions, the containment strategy employed in the case of SARS required ‘nationwide total mobilization’ led by the central government (Chung, 2016, p. 112). Chung concludes that the total mobilisation that followed the sacking of China’s Health Minister in April 2003 resulted in the central government containing the SARS virus within two months. By this standard, the extreme contingency of the COVID‐19 outbreak should limit discretion for local authorities. The COVID‐19 policy was extremely urgent, treated by Beijing as a wartime response, leaving little room for local discretion according to Chung’s (2016, p. 91) analysis of extreme contingencies. But evidence from Nancun village, Hebei Province,^2^ gathered during the peak of the COVID‐19 crisis from January to April 2020 shows the importance of local disease control initiatives and divergent emergency responses.

The politically sensitive COVID‐19 emergency response policy was all‐encompassing in scope (applied to all local units) and was therefore expected to strongly restrict discretion (Chung, 2016, p. 90). In January 2020, the Politburo Standing Committee, China’s powerful executive, established the Central Epidemic Response Leading Group, which represents strong centralised control. However, beyond the development of national master plans for COVID‐19, there were degrees of policy discretion at subnational levels that enabled localised and context‐specific responses to the virus within China’s strict bureaucratic hierarchy. Drawing on qualitative field research, this article illuminates several key elements of local disease control initiatives and finds that emergency response involves some decentralised control, with local authorities adapting national policies to meet local needs. Local discretion in governance and technical responses to COVID‐19 are found, with national bureaucratic procedures being adapted or suspended to meet the challenges of the disease, and a variety of subnational actors and resources being effectively mobilised. The authors observe forms of local discretion in the enforcement of lockdown policy, travel restrictions, quarantine, testing, track and trace, supply chain management, information dissemination, discipline, mobilisation of non‐state actors, and the reassignment and evaluation of personnel.

Our empirical findings broadly support the ‘encompassing‐governance‐urgent’ policy dimensions of an extreme public health emergency such as SARS or COVID‐19, but we argue that within a constrained, coercive environment there are local forms of policy discretion and divergence that should be documented. For a total mobilisation policy response to work, coordination with subnational units is required, and the nature of subnational level policy responses matter.

This article proceeds as follows: Section 2 outlines the study’s research context and methodology. Section 3 explores the three‐staged response to COVID‐19 in Hebei Province, and Section 4 provides an in‐depth discussion of local discretionary measures in Nancun village. Section 5 examines the complexities of local public compliance during different stages of lockdown. Section 6 reviews China’s COVID‐19 containment strategy in the context of a decentralised response model. Section 7 concludes with reflections on central‐local relations based on evidence of government control measures and local discretion.

## 2 RESEARCH CONTEXT AND METHODOLOGY

Studies suggest that collaborative crisis management often follows the bottom‐up principle of ‘disaster subsidiarity’ with local authorities leading responses, and even in top‐down centralised systems such as China and Vietnam there is a need for central‐local coordination to manage complex emergencies (Parker et al., 2020). There is evidence of mismatched decentralisation policies in China where, for instance, national‐level environmental regulations are deliberately ignored by local governments because of the disincentives and losses accrued by local businesses (van der Kamp et al., 2017). Local disobedience of this kind has been largely absent or hidden from the COVID‐19 response, although discretion is exercised by local governments on the front line of this wartime policy. Given the need to mitigate the potential damage to regime legitimacy caused by mismatched policies, a model of differentiated response from central and local governments has emerged, where the cost of action is weighed against the need for responsiveness (Cai & Zhou, 2019, p. 333). In a central government move to curtail ‘upward targeting’ by an anxious public during the COVID‐19 emergency (Chen & Cai, 2021), surveillance and ideological messaging intensified in early 2020 (Moynihan & Patel, 2021). If problems exposed by social media or petition usually signal local‐level governance failures that enable Beijing to hold local authorities to account and evaluate performance (Cai & Zhou, 2019), then the all‐encompassing urgent COVID‐19 response will have created short‐term blind spots at local levels that will likely generate residual pressure and upward targeting in the future.

Oberlander (2020) notes that responses to COVID‐19 are shaped by familiar social and political institutions, though in China’s fragmented authoritarian system, a variety of subnational institutions have been developing emergency response efforts to meet local contextual conditions. Fragmented authoritarianism, first elucidated by Lieberthal and Oksenberg (1988), denotes a system in which central policy is malleable, reflecting a range of political and organisational interests, and is ‘governed by incremental change via bureaucratic bargaining’ (Mertha, 2009, p. 996). Despite the invasive monitoring of local authorities by the central government, corruption and policy deviation remains difficult to identify in China’s mandate system, known to produce relative standards and shifting priorities that increase the information burden (Birney, 2014). After the global outbreak of SARS in 2003, the Chinese central government identified shortcomings in national emergency management and moved to restructure the entire system, with varying degrees of success (Lu & Xue, 2016). The focus on emergency response may have led to improvements in the national system; however, the similarities between the COVID‐19 crisis and SARS in 2003 shows that the underlying challenges of China’s ‘rule of mandates’ system (Birney, 2014) are politically complex and unresolved.

On 25 January 2020, the Politburo Standing Committee established the Central Epidemic Response Leading Group (中央应对新型冠状病毒感染肺炎疫情工作领导小组), headed by Vice President Li Keqiang and deputised by Wang Huning.^3^ Party discipline is central to the ideological dimension of emergency response, with governance including expectations of collective public sacrifice and loyalty. On 3 February 2020 President Xi Jinping branded China’s fight against COVID‐19 the ‘people’s war’ and insisted that Communist Party committees and governments at all levels follow the directives of the Party Central Committee and their epidemic response advisors (Xi, 2020). During the peak of the outbreak in February the Central Epidemic Response Leading Group held meetings every two to four days. Given the ‘encompassing‐governance‐urgent’ nature of COVID‐19 policy (Chung, 2016), while the central government preferred total control it needed to coordinate with subnational units. The COVID‐19 policy therefore had a dual nature, involving tangible resource allocation and governance (political and ideological) imperatives that demanded high levels of compliance from local political units.

Hebei Province is part of the Beijing‐Tianjin‐Hebei region located in the northeast known as the ‘capital circle of China’ (Yang et al., 2018). The number of recorded COVID‐19 cases in Hebei on 27 April 2020 was 328, compared with 68,135 cases in Hubei Province (including the provincial capital Wuhan in central China). Qicun township in Hebei is the site of a 2018 pilot project for the improvement of the rural governance called Three Forces and Three Types of Governance (三力三治). The idea was to build a functioning platform for cooperation (共建共治共享) between the local government, rural autonomous organisations and CCP members, which turned out to be a timely initiative given the unexpected COVID‐19 outbreak. Nancun is the largest village in Qicun, with some 4000 residents accounting for nearly one‐third of the total township population. There were no recorded cases of COVID‐19 in Nancun during the period of research (January to April 2020) despite some people returning to the village from the Chinese epicentre in Wuhan to celebrate the Lunar New Year.

Participant observation in Nancun took place during the first two months of lockdown in 2020 when travel restrictions were in place and families were ordered to stay home. By late February 2020, when the national epidemic was contained and Nancun village relaxed its lockdown restrictions, it was possible to conduct interviews. The authors interviewed village committee members, Party members, healthcare workers, businesses and residents in Nancun village from February to April 2020, and secured interviews with the Qicun township government in February 2020. Informed consent was obtained from all participants in Nancun, and government officials from Qicun township agreed to contribute to the study because they believe their work to be ‘worthy of publicity’ (Interview: Qicun township government, 28 February 2020). Qicun township officials agreed to travel to Nancun to participate in interviews on 28 February 2020. Participants included the head of the township government (who also serves as the Deputy Party Secretary), the director of the publicity department and various other officials in supporting roles. All participants in the research could speak freely, under condition of anonymity, about their experiences in responding to the COVID‐19 crisis. Interview questions focused on local response strategies and approaches, and the ways in which various groups were mobilised to contain the transmission of COVID‐19 including local authorities, Party cadres, physicians and healthcare practitioners, social organisations and volunteers. To crosscheck and give further context to these interviews, unpublished government documents and reports were examined along with media coverage of the crisis.^4^

## 3 THREE‐STAGED RESPONSE TO COVID‐19 IN HEBEI PROVINCE

The responses to COVID‐19 in Hebei Province can be separated into three stages. Stage 1 is from 31 December 2019, when the outbreak of COVID‐19 in Wuhan was first reported, to 23 January 2020 when the Qicun town government was instructed by the subnational county government to monitor all migrant workers and students returning from Hubei Province (Interview: Qicun town government, 28 February 2020). As a local leading group for COVID‐19 prevention had not yet been established at this early stage, all the track, trace and monitor work was carried out by local organisations without formal supervision from the township government. This meant that if locals failed to disclose that they had recently returned from Wuhan, the village cadres ‘would not put their fingers into another’s pie’ (Interview: Nancun village cadres, 24 February 2020). Residents from Nancun recall that two university students who came back from Wuhan had been seen wandering around the village before the Lunar Festival. If the students were subsequently diagnosed with COVID‐19 ‘the whole village would be finished’ (Interview: Nancun residents, 22 February 2020). This early‐stage response shows that in the absence of central government command, which takes precedence, subnational authorities had the flexibility to enact localised and context‐specific policy responses to the virus.

Stage 2 is from 24 January 2020 to late February 2020, the most intensive period of COVID‐19 control when the town government formed a leading group to combat the virus (see also Section 4). To prevent the spread of COVID‐19 an integrated approach involving local physicians and healthcare workers, autonomous organisations and schools was implemented by town authorities. During this period all transport in Qicun was blocked, public screenings for the virus were held five times per month and surveillance was focused on locating students and all other returnees from Wuhan based on a name list from the County Public Security Bureau. These bureaus can accurately track people who have passed through Wuhan by plane, train or automobile using the Skynet system (天网系统), the ID‐based ticket booking system and mobile positioning system.

Stage 3 is from 1 March to 30 April 2020 when the epidemic was contained, and lockdown restrictions were eased. Travel restrictions were lifted, and people could come and go from the village without permission from the government. Surveillance and health screening had ended by March because zero COVID had been achieved, but the working groups were kept on standby, prepared to respond if any positive COVID cases were identified. People could also return to work. Universities decided to remain closed until September.

Stopping the spread of COVID‐19 infection in rural areas is one priority of epidemic prevention that has been repeatedly stressed by national leaders. For example, Xi Jinping delivered a speech in Beijing on 10 February 2020 declaring that the government ‘must give due consideration to both urban and rural areas, so that rural epidemic prevention is not left behind’ (Zhang, 2020). At the initial stage of the COVID‐19 outbreak, millions of migrant workers (农民工), university students and sojourners were returning from China’s megacities to celebrate the Lunar New Year. According to the National Bureau of Statistics (2019), there were 564 million rural residents in China as of the end of 2018, accounting for over 40% of the county’s total population.^5^ In 2018 there were an estimated 180 million migrant workers in China, in addition to nearly 1.5 million university students in Hubei Province (the picturesque capital Wuhan is a popular study destination), making the Lunar New Year homecoming one of the largest annual transmigrations in the world (Dong, 2019; Ministry of Agriculture and Rural Affairs, 2018). This scale of movement poses immense challenges for public health officials during a pandemic. The complexity of epidemic prevention in rural areas with relatively poor infrastructure and suboptimal medical conditions increases the risk of disease transmission. National Health Commission analysts who track the daily spread of COVID‐19 suggest that the transmission of the virus in China’s vast countryside has been reasonably well contained, with no large‐scale infections outside of Wuhan and Hubei Province.^6^

## 4 SYSTEMATIC LOCAL RESPONSE TO COVID‐19

China entered its second and most intensive stage of COVID‐19 response following a public announcement on 20 January by Zhong Nanshan that the disease could spread from person to person (Zhong, 2020). Zhong, a retired pulmonologist and former president of the Chinese Medical Association, was China’s leading figure in the 2003 fight against SARS. Now in his eighties, Zhong was called upon to lead the COVID‐19 epidemic response in Wuhan. He is one of China’s most respected medical experts and has the trust of the public.

In China, townships are the lowest administrative units with emergency response powers (属地管理) as stipulated in the 2007 Emergency Response Law. Below the township government there is no administrative unit at the village level (Wang, 2015). The grassroots regime from which cooperative responses to crises emerge is located at the intersection of state and society, connecting township governments with quasi‐administrative and Party branch committees at the village level. On 23 January 2020, Qicun township established a leading group consisting of civil servants and doctors to coordinate the activities of seven core groups:

- The office of the leading team (领导小组办公室).
- The comprehensive coordination group (综合协调组).
- The epidemic disposal group (疫情处置组).
- The material support group (物资保障组).
- The publicity guidance group (宣传引导组).
- The emergency prevention team (应急防控组).
- The supervision and inspection group (督导检查组).

Each group performed specific functions and cooperated with other groups to carry out core work in epidemic prevention as well as public outreach and related activities. At least 20 cadres from the Qicun township government along with local health centre staff were seconded to the seven groups, and at the time of interview ‘none of them have had a day off since’ the groups were formed (Interview: Qicun town government, 28 February 2020). Respondents from Qicun did not disclose any budgetary information, though they did claim that they stockpiled virus prevention materials and personal protective equipment when cases of infection in Wuhan were reported.

The argument that ‘formal organization charts often hide as much as they reveal about where real power lies’ in China’s authoritarian system is still valid today (Saich, 2011, p. 142). Party secretaries wield considerable direct and indirect power throughout all of China’s institutions at virtually every level. China’s administrative state may be fragmented, granting individuals ‘immense capacity to circumvent formal regulations’ (Saich, 2011, p. 143), but this capacity is limited in the context of the COVID‐19 emergency, where subnational authorities are routinely reminded of the consequences of dereliction of duty or poor performance. China’s complex system of policy networks and competing loyalties is compounded by the blurred lines of accountability, with local officials wearing multiple hats but ultimately serving party interests. For instance, the administrative head of Qicun township, who serves the parallel (and superior) role of Deputy Party Secretary, is formally responsible for epidemic prevention work overseen by the leading group.^7^

Nancun village authorities formed their own COVID‐19 response group led by the local branch Party secretary and supported by 11 village cadres, village doctors, and school headmasters at primary and secondary levels. This organisational system proved effective in part because of the 2018 implementation of ‘grid governance’ (网格化治理). Grid governance, a significant part of the CCP’s post‐2004 efforts to improve administrative efficiency, is a ‘grassroots’ multi‐actor network governance structure that operates at subnational level, where personnel assignments and public services are allocated based on grid boundaries (Tang, 2020). In 2018 Qicun township adopted a grid governance model that divides rural areas into a series of grids that select their own ‘grid members’ (网格员), most often Party cadres, to coordinate decisions and policies on specific local issues (Interview: Nancun village cadre, 24 February 2020). On 23 January 2020, after a video meeting held by the town government, Nancun immediately issued a notice that new epidemic prevention centres must be established along grid lines, showing the surveillance potential inherent in this system. In each prevention centre two Party cadres and one village doctor coordinated their responses to COVID‐19 with grid members. By the second and most intensive stage of China’s outbreak Nancun village was divided into six epidemic prevention centres.

Following are some of the findings of our qualitative field research and the key elements of the local discretionary measures adopted in Nancun village in response to the COVID‐19 emergency during the study period.

### 4.1 Operational mechanisms

In the complex and sometimes fraught process of epidemic prevention, local‐level authorities adopted a highly mobilised operational mechanism that combined the formal political resources of the state with informal resources drawn from the village constituency. The operational mechanism observed by the authors was designed to control population flows. From the outset of the COVID‐19 outbreak in January 2020, many measures were adopted to achieve this goal, such as imposing traffic restrictions, cancelling almost all Lunar New Year gatherings and celebrations, and appealing to all residents to self‐isolate by staying in their homes. The only way to achieve high levels of compliance was to ensure adequate supplies of food, medicine and basic goods to avoid panic and disorder. The local Party‐state apparatus also deployed various forms of surveillance and propaganda to manage the villagers. Village elections have, in procedural terms, improved considerably since the 1990s but governance is still often shaped by the dynamics of village politics in which elite factions vying for the office of Party secretary control resources and wield coercive power over local populations (Yao, 2017).

### 4.2 Population screening

Nancun village authorities carried out a series of household inspections from February to March 2020. During each inspection, a village cadre along with a Party member or a volunteer in protective equipment surveyed the population using household registration data and reported their findings to the township government. The first public screening for COVID‐19 in February prioritised residents who had recently returned from other cities, especially Wuhan. This group of at‐risk returnees were sent to designated isolation centres and their families were quarantined at home. Village authorities decided to put warning signs on the doors of all isolating households to discourage visitors. The signs provided the contact information of the village cadres and doctors in charge of the family being quarantined (Interview: Nancun village cadre, 24 February 2020). Two follow‐up investigations took place focusing on priority individuals who had returned to Nancun for the Lunar New Year celebrations. Two village‐wide screenings were also conducted in this period to crosscheck the household registry data. The aim was to confirm the number of people in each family, to record any travel that had taken place from December 2019, to identify any specific links to Wuhan (study or work related), and to screen for any flu symptoms (Observations in Nancun village, February 2020). Nancun’s grid governance model is contentious from a privacy and freedom perspective (Zhao, 2014), though it seems to have enabled a dynamic and effective response to the COVID‐19 epidemic.

### 4.3 Travel restrictions

By 25 January 2020, all 21 villages in Qicun town had imposed travel restrictions and blocked traffic, leaving only one entry and exit checkpoint to restrict access in each village. Nancun opened two corridors with checkpoints connecting with other villages. In the Chinese tradition, 25 January is an important time to visit relatives after the Lunar Festival. When the traffic blockade was imposed on such an auspicious date, village residents quickly grasped the seriousness of the epidemic (Interview: Nancun villagers, 25 February 2020). There was naturally some discontent; however, public acceptance of the lockdown may have been aided by memories of the 2003 SARS outbreak (Interview: Nancun villager doctor, 29 February 2020).

Traffic checkpoints were highly visible, with detailed signposting and uniformed staff equipped with infrared thermometers, disinfectant sprayers, tables and chairs, tents, pillows, bedding, provisions, electric heaters, and other materials. At each checkpoint there was a village cadre and a Party member or volunteer on duty 24 hours a day. Township level cadres inspected the village checkpoints periodically every day. If they found that no one was on duty at the checkpoint, the village committee—which was responsible for upholding lockdown rules and accountable for any outbreaks—faced disciplinary measures. During this stage of the COVID‐19 response residents from other villages were not permitted to enter Nancun. Residents of Nancun who wished to travel had to follow a strict system of ‘checking, asking and registering’ (一查二问三登记) before receiving a travel permit from the village committee (with approval from the township). The only grounds for obtaining a permit was to visit a hospital, and applicants needed a letter from their physician confirming their health condition.

### 4.4 Daily reporting

Village authorities immediately reported any people returning to the village to their superiors at town level. From January to April 2020 villages were also required to complete daily reports by 11am and return the information to the Qicun town government, with emphasis on arrivals from Wuhan. Failure to disclose and report was considered a serious dereliction of duty. If the village was found to have deliberately concealed information, the Party branch secretary and their deputies faced automatic dismissal. If the relevant data was not reported in a timely manner, village cadres received a warning and may have faced disciplinary measures (Report: Qicun township government, 3 May 2020). The authors learned of at least one case of immediate dismissal for failing to report. The head of Qicun township received news of an arrival from Wuhan early one morning, but when he contacted the Party secretary in the village concerned to check the details, he found that the secretary knew nothing about the matter. This suggests that the township government had set up an additional information gathering network for real‐time monitoring of the changing situation. The head of the township government confirmed that ‘we have our own information channels’ (Interview: Qicun township government, 28 February 2020), which indicates that the township created a new network to gain detailed information rather than relying on the reports from village authorities and cadres. The village‐level Party branch secretary was immediately dismissed for failing to report a person returning home from Wuhan (Interview: Qicun township government, 28 February 2020).

Expulsion from the Party also means losing one’s cadre status. In rural China, the Party branch secretary oversees the rural collective economy, deciding how to distribute land and manage rural enterprises, which can be very lucrative (Zhang, 2018). In addition to the economic benefits, the status of village cadre can also bring long‐term political benefits. Since the 1988 Organic Law (村民委员会组织法), village committees across China have been elected by villagers with varying degrees of legitimacy and propriety (O’Brien & Li, 2000). Local Party branches tend to control nominations, however, and town governments make their candidate preferences known, creating dependencies and distortions tied to local patronage (Interview: Qicun township government, 28 February 2020).

### 4.5 Quarantine and mandatory isolation

At the start of the outbreak the government rented hotels and student dormitories and converted them into quarantine centres. People returning from Hubei Province were directed to isolate at a designated quarantine centre for 14 days and then self‐isolate at home for a further 14 days. Village doctors were responsible for preliminary identification, and after reporting symptomatic patients and suspected COVID‐19 cases to the town government, the health centre arranged special medical staff and vehicles to transfer patients to the quarantine centre for 14 days. Only then did testing and tracing begin. Once discharged, people isolated at home with their family for an additional period of 14 days. The town health centre provided thermometers, disinfectants and supplies for the village doctors, and village cadres distributed a list of precautions and guidelines for families in isolation. For instance, isolating households received an information chart (信息表) to record their physical condition every day, as well as a service card (服务卡) to request provisions and essential items needed during lockdown. Village cadres worked with the local police to ensure the safe distribution of goods. The wellbeing of families in isolation was also given consideration, with counselling services ostensibly offered by village authorities, but there is no data available about this service.

### 4.6 Managing supply chains

One formidable challenge during lockdown restrictions in the first two months of 2020 was ensuring an adequate supply of medical resources and staple goods. Supply chain disruptions bring the risk of scarcity and inflation, leading to fears of public riots. Some villages in Qicun experienced price hikes in mid‐February 2020, and in one example when the price of flour suddenly increased in a supermarket locals saw this as a sign of shortages and started panic buying. The town government quickly intervened and revoked the store’s business licence, sending a clear message to other local supermarkets which then maintained pre‐lockdown prices for staple products (Interview: Qicun township government, 28 February 2020).

On 17 February 2020 the town government announced a plan to ensure the supply of daily necessities within their jurisdiction. Qicun authorities had access to the county distribution centre that was temporarily set up to meet demands for medical supplies, sanitary products, and food. The township appointed a logistics team to work directly with 10 supermarkets in their jurisdiction while liaising with county distributors and village supermarkets. Travel restrictions remained in place, but the town government created a ‘green channel’ (绿色通道) for the supply of daily necessities. Staff from one supermarket recalled that daily shipments reached the villages, with designated delivery drivers approved by village cadres supplying stores to limit physical contact (Interview: Nancun supermarket, 27 February 2020).

A degree of market intervention was required during the epidemic. Apart from pharmacies and supermarkets that supply essential goods, all other businesses faced temporary closures. Pharmacies and supermarkets that operated during the lockdown were granted special permission from the town government. Shop entrances had screening and testing tables to check the temperatures of customers, and a record was kept of all customers so they could be identified and contacted in the case of an outbreak. All shoppers were required to wear masks. The town government implemented a weekly market monitoring system to try to maintain the supply of essentials and manage the demand for grain, meat and vegetables. Supermarkets were advised to replenish goods to avoid panic and the impression of shortages (Report: Qicun town government, 3 May 2020). Pharmacies in Senzhong and Qicun were tasked with the daily monitoring of the market supply and demand for preventive equipment such as masks, thermometers and disinfectants. The town government supervised the prices and inventory of necessities to ensure stability. Staff from one village supermarket reported that in February people posing as regular customers casually enquired about the prices of various commodities, only to report suspected price hikes to the town government. At the time of research there were stories and rumours of supermarket closures in Qicun township (Interview: supermarket owner from Beicun village, 27 February 2020).

## 5 PUBLIC COMPLIANCE

The leading groups working to combat COVID‐19 continue to follow the basic outlines of the government’s organisational structure. Since May 2020, there has been a redeployment of staff and some reorganising of processes and procedures, but the epidemic response groups and their constituent members are today still upwardly accountable to the relevant government departments. After the COVID‐19 outbreak there was a risk of panic spreading among communities; ‘pestilence is coming’ became a common refrain used by village elders in their daily conversations, referencing the horrors of the plague (Interview: Nancun residents, 22 February 2020). In the era of smartphones and instant communication, villagers can quickly disseminate news of varying degrees of reliability about the spread of infections. Doctors in the township health centre were equipped with protective clothing, goggles and masks when transferring symptomatic patients, and while these were preventative measures the optics of these scenes caused panic among some villagers who assumed that patients were infected with COVID‐19 (Interview: Nancun village doctor, 25 February 2020).

On 18 February 2020 stories began to circulate in Nancun village about a student who returned from Wuhan and still had a fever after 14 days of isolation. As the news of this unconfirmed COVID‐19 case spread, the severity of the epidemic grew in people’s minds. People in Qicun town began to phone relatives in Nancun to find out whether the rumours were true (Interview: Nancun villagers, 23 February 2020). Tensions rose as Nancun village went into total lockdown; the streets were empty, and families began to accuse and blame each other of breaching the lockdown rules, for example going outside to play mahjong (Interview: Nancun villagers, 25 February 2020). It later emerged that one resident who visited a doctor in Cangzhou City, Hebei Province, before the Lunar New Year and self‐isolated after returning to Nancun on 18 February 2020 had been visited at home by some villagers who were unaware of the situation at first and had inadvertently broken the rules (Interview: Nancun village cadre, 26 February 2020).

The false alarm in Nancun meant that neighbouring villages such as Beicun took even greater COVID prevention precautions and residents avoided anyone from Nancun, even close friends and relatives (Interview: Nancun villagers, 23 February 2020). The Party secretary of Nancun took the pragmatic view that ‘there is no need to dispel the rumours surrounding the village’ because, in the short term at least, it makes it ‘easier to prevent people from coming to our village’ (Interview: Nancun Party secretary, 26 February 2020). Within the village the opposite approach was taken by cadres as the rumours were damaging social relations and undermining trust. Village authorities requested permission from the town government to publish the personal information of families in isolation. The response from Qicun was that there were legal concerns, although as the purpose of publishing the private information was to protect the public during an emergency, it was therefore deemed permissible (Interview: Nancun village cadre, 26 February 2020). Following this decision all information related to people in isolation was published in an official village WeChat group.

Township authorities spent considerable time and energy trying to contain the threat of disinformation during the peak of the COVID‐19 outbreak. Qicun officials routinely checked epidemic information broadcasts, inspected public spaces to ensure public health banners^8^ were visible, checked that up‐to‐date leaflets were circulated, and monitored the official COVID‐19 WeChat groups in each village. The town government carried out random inspections and stipulated that if more than three people in any given village claimed to be unaware of virus prevention measures, the village cadres would face disciplinary measures. Fearful of negative performance reviews and career prospects, villages cadres turned to patriotic appeals online and, in more traditional style, belted out daily broadcasts over loudspeakers. ‘Big horn projects’ (大喇叭工程) are a familiar feature of rural life under China’s communist regime, with orders and instructions concerning all sorts of public (or more likely Party political) interest issues piercing the airwaves. The ‘horns’ and loudspeakers, largely removed by Xi Jinping’s predecessors, seem to have been revived as a governance mechanism and were used with regularity during the COVID‐19 crisis.^9^ Many village residents played their part by opening their front doors to listen, but admitted that they couldn’t really hear the announcements (Interview: Nancun villagers, 23 February 2020).

During the SARS outbreak in 2003, many villagers in Nancun questioned the lockdown policy. But during the COVID‐19 emergency response the village committee did not face much opposition—many villagers in Nancun supported the lockdown approach. From January to April 2020, the researchers only learned of one breach of the COVID‐19 lockdown. An intoxicated man from Nancun found his way to neighbouring Beicun village and was apprehended by police. The next day Nancun residents spoke with disapproval about the matter and showed little sympathy for the man who was in police custody. Access to medicine was a different matter, however, with villagers showing a greater degree of flexibility, and some rule bending occurred. Relatives of village doctors, who are responsible for managing pharmacies and controlling prescriptions, seemed to be able to get easy access to medicines and a grey market emerged (Interview: Nancun villagers, 15 February 2020).

## 6 CHINA’S DECENTRALISED EPIDEMIC RESPONSE MODEL

China’s effective containment strategy for COVID‐19 explains the sub‐exponential growth of cases (Maier & Brockmann, 2020) and limited large‐scale outbreaks across the country. China’s authoritarian approach is contentious, but by mobilising townships and villages the government saved time and resources. Rural areas made significant contributions to epidemic prevention, and the variety of administrative responses show the importance of decentralised responses in China (Ma, 2020).

During the 2003 SARS outbreak researchers concluded that local authorities and social organisations were under the control of China’s powerful central state, playing passive, cooperative roles with limited participation in epidemic response (Geng & Hu, 2011). Grassroots mobilisations were led by the interventionist state to achieve quick results and bolster legitimacy. In 2020, facing another epidemic potentially linked to illegal wildlife trade and irregular practices at fresh food markets (van Staden, 2020), a similar response mechanism was adopted. The model can be characterised as movement‐type governance, a spinoff of the Mao‐era ‘mass campaign’ modified during the fight against SARS and COVID‐19 with far‐reaching impacts on rural village life. Everyday routines and rituals were affected; weddings were postponed, funerals were scaled back, schools were closed, and travel was banned (Hu, 2011). Special emphasis was placed on differentiating between native (本村人) and non‐native (外村人) villagers as an administrative means to identify and monitor the local population, but also potentially as a ploy to promote models of civic virtue.

The village leading group in Nancun recruited 42 students and Party members as volunteers, a mobilisation that did not happen during the SARS outbreak in 2003. The volunteers assisted in population screening, data collection and daily reporting, which alleviated the pressure on overstretched village staff. Local capacity limitations mean that human resources, infrastructure and facilities are underdeveloped in rural China. Only two village cadres in Nancun are under 40 years old, and as they are considered computer literate, this placed a heavy workload on their shoulders. The two computer operators felt anxious during the COVID‐19 crisis, when data was requested daily, and the work of university student volunteers helped them to cope with the demands of reporting (Interview: Nancun village cadre, 24 February 2020).

Village doctors rallied to the cause, being motivated to participate in the epidemic prevention effort without any additional financial support. In China’s villages there are many so‐called barefoot doctors (赤脚医生), a reference to part‐time paramedics trained in simple techniques of diagnosis and treatment who work as unpaid volunteers under no obligation from the government and village committee. From observations in the field, it was clear that village doctors participated on the front line of the fight against COVID‐19, including monitoring and recording temperatures of people in isolation even when there was no personal protective equipment available (Interview: Nancun villagers, 22 February 2020). The Party branch secretary claims that ‘these doctors were reluctant to attend village meetings in the past; however, this time was different, they joined all the video meetings without hesitation’ (Interview: Party branch secretary, 26 February 2020). The village doctors’ physical and online participation stemmed from a moral responsibility to the community.

The novel response to COVID‐19 is further evidence by villages exercising a certain degree of autonomy over policy interpretation. During lockdown, the township government stipulated that village residents can only travel ‘for major events’ (Report: Qicun town government, 3 May 2020). At first the village leading groups did not know how to determine what a major event was, and simply prohibited everyone from leaving Nancun. Upon reflection it was decided that only sick villagers with certificates from their doctor could leave the village to seek further testing and consultation at hospital, and should provide a hospital certificate on their return. The practical decision to allow travel exceptions on medical grounds was subsequently adopted by neighbouring villages, followed by the Qicun township government (Interview: Nancun village cadre, 26 February 2020).

Rural lockdown measures were strict and widely adhered to, though not all the directives of the township government were enforced. Cases of corruption and collusion occurred during lockdown. For instance, one mah‐jong hall in Nancun, whose owner was a close friend of the deputy director of the village committee, remained open in February 2020. When the township government came to routinely inspect the village, the deputy director, who was known to play mah‐jong every day except when he was on duty at the village checkpoint, warned the mah‐jong hall so that it could close before inspectors arrived (Interview: Nancun villagers, 26 February 2020). By contrast, the Party branch secretary Gu Xingkai worked tirelessly during the peak of the COVID‐19 crisis. Secretary Gu’s only son died unexpectedly before the Lunar New Year (unrelated to COVID‐19), and news quickly spread of his personal tragedy as well as his unwavering commitment to his duty. He became a symbolic figure in the fight against the epidemic and received significant media coverage throughout Hebei Province (Lin, 2020).

The utilisation of new technologies and artificial intelligence has strengthened the governing capacity of the Chinese state. Vast quantities of data can be obtained by the government, including information about people visiting or passing through Wuhan, their whereabouts, activities, and contacts. WeChat, China’s most popular social media and communication platform, can be used as a tracking technology, producing heat maps for crowd control and the monitoring of individuals, vaguely referred to as persons of interest (Feldstein, 2019, p. 44). The increasing availability of big data, in the hands of authoritarian and democratic regimes alike, has led experts to warn of a ‘road to digital unfreedom’ (Feldstein, 2019). The potential for repression and control is obvious, though in the context of an epidemic such a surveillance capacity may be advantageous and even welcome. Access to big data and pattern recognition software would allow local governments to take more targeted measures to prevent the transmission of COVID‐19 by, for instance, monitoring those in isolation and screening the population. Inevitably some people are reluctant to disclose their whereabouts or travel itineraries and refuse to participate in contact tracing when there is mass panic about a highly infectious disease. Township and village authorities faced problems when attempting to screen returnees from Wuhan (Interview: Nancun village cadre, 26 February 2020); however, it seems that tracking technologies helped fill the gaps.

A confluence of factors, including the use of policy discretion by local authorities, strengthened central state authority during the COVID‐19 crisis. Xi Jinping attached great importance to the ‘people’s war’ against the coronavirus, striving to showcase the adaptive and responsive nature of the central government in a bid to legitimise one party rule. Once the Politburo announces an ‘important instruction from Xi Jinping’, it becomes a top priority for the government. President Xi is thought to be a true believer in the communist cause, having ‘red genes’, and is more assertive than his predecessors. Observers such as Pei (2020) contend that the centralisation of power exposes the CCP to risks, but past disasters such as the 2008 Sichuan earthquake show the will of the CCP to turn tragedy into triumph through intensive public relations campaigns focused on positive factors such as the speed of relief efforts and the strength of public solidarity (Schneider & Hwang, 2014). Wuhan now has a COVID‐19 museum and exhibition hall in a repurposed hospital that documents the terrible struggle against the virus and celebrates the triumph of the CCP, showing the continued salience of positive propaganda in China. Here we see a manifestation of ‘contingent destiny’, where China’s return to the centre stage of world politics is possible only if central party leadership is maintained (Breslin, 2019, p. 29).

## 7 CONCLUSION

Since 2012 the Chinese Communist Party under President Xi Jinping has reasserted central political and ideological control while tightening its grip on key bureaucracies. Yet, in the context of Beijing’s centralised emergency response measures and policies, this article finds degrees of local policy discretion that enable context‐specific responses to COVID‐19 in China’s fragmented authoritarian system, where competing interests render central policy malleable. Evidence from Nancun village in Hebei Province illuminates some key elements of decentralised disease control initiatives, with local governments adapting national policies to meet local conditions while mobilising a variety of actors and resources.

Within China’s centralised unitary system, dynamic centrifugal forces continue to challenge central government authority. As central‐local relations evolve, a complex balance is struck between control and discretion based on the scope, nature, and urgency of policy (Chung, 2016, p. 90). When faced with extreme contingencies such as the 2002–2003 SARS emergency or the current COVID‐19 emergency it is logical for Beijing to seek to limit discretion for local authorities. At the same time, central‐local coordination is required for an effective COVID‐19 total mobilisation policy response to work. In the context of Chung’s (2016) ‘encompassing‐governance‐urgent’ policy characterisation of an extreme public health emergency, our evidence from Nancun gathered during the peak of China’s COVID‐19 crisis from January to April 2020 shows that fragmented forms of policy discretion influence local disease control initiatives and emergency responses within China’s authoritarian system.

The top‐down central response from China’s leaders in early 2020 was to declare a ‘people’s war’ against the virus and to limit damage to regime legitimacy by making containment the government’s top priority. Degrees of subnational variance exist across China’s vast rural areas and countryside, where there are structural challenges and capacity issues that include shortages of qualified medical personnel and resources. When the first wave of COVID‐19 infections spread during the Chinese Lunar New Year, with some five million people leaving the virus epicentre in Hubei Province before a travel ban was imposed, the vulnerable townships and villages in this study contained the virus by undertaking three phases of emergency response: hard lockdown, with track and trace used to identify all returnees during the holiday period and to enforce quarantine measures (official report of the outbreak in Wuhan on 31 December 2019 to 23 January 2020); intensive surveillance period, with a formal leading group established by the township and village government to combat the virus (24 January to end February 2020); and easing of lockdown restrictions, when the 21 villages of Qicun township recorded zero infections (March to April 2020). Local authorities had managed the influx of returnees from various parts of China during the Lunar New Year celebrations.

This rapid containment is a significant indicator of the importance of local responses in the fight against COVID‐19, where constrained but still significant local discretion exists in most major policy realms. Local resistance to central mandates in China, resulting from ‘mismatched decentralization’ policies (van der Kamp et al., 2017) have been curtailed during the extreme COVID‐19 emergency response. Discretion is nevertheless exercised by subnational governments on the front line of China’s wartime public health policy. Multilevel and differentiated government responses to citizen pressure and public emergency in China serve as an important benchmark for comparative studies of political legitimacy in both democratic and non‐democratic contexts. The exceptional COVID‐19 national emergency enabled the central government to increase its power over local authorities and its leverage over citizens, boosting performance legitimacy in the short term. The challenge now is how to continue introducing technologies and controls into the decentralised grid governance system to improve responsiveness to public health and other emergencies. And, importantly, how to do this while simultaneously protecting privacy and improving feedback channels between different levels of government to avoid incentives for local cover‐ups by officials wary of performance evaluations and career prospects.

## CONFLICT OF INTEREST

No conflicts to declare.

## Data Availability

Qualitative data was obtained in the field in early 2020 with the consent of participants. The data is not publicly available due to privacy or ethical restrictions. Further information about research methods and data gathering is available on request from the corresponding author.

## Footnotes

^1^
The first cases of SARS emerged in mid‐November 2002 in Guangdong Province, China. SARS was recognised as a global threat in March 2003 after the virus spread to countries in Asia, Europe, North America and South America: see https://www.cdc.gov/about/history/sars/timeline.htm.

^2^
Nancun village, population 4000, is the largest village in Qicun town, Hebei Province.

^3^
Additional members are Ding Xueyang (Political Bureau of the Party Central Committee), Sun Chunlan (Vice Premier of the State Council), Huang Kunming (Director of the Publicity Department of the Party Central Committee), Cai Qi (Political Bureau of the Party Central Committee and Secretary of the Beijing Municipal Party Committee), Wang Yi (Minister of Foreign Affairs), Xiao Jie (Secretary General of the State Council) and Zhao Kezhi (Minister of Public Security).

^4^
Official Chinese‐language documents include the Novel Coronavirus Prevention Programme in Qicun, the Notice on the Implementation of Key Measures for Epidemic Prevention, the Supply and Distribution Plan for Necessities in the Jurisdiction of Qicun, and the Summary of Novel Coronavirus Prevention in Qicun.

^5^
Rural residents are identified according to their household registration, or hukou (户口). Data from the National Bureau of Statistics (2019).

^6^
Cases of COVID‐19 were reported daily from 11 January 2020 on the National Health Commission’s website: http://www.nhc.gov.cn/xcs/yqtb/list_gzbd_29.shtml.

^7^
In China’s parallel administrative system, the Party secretary is always first in command.

^8^
One public epidemic prevention banner read ‘running around today, in hospital tomorrow’.

^9^
See Li (2020) for an analysis of loudspeakers in the Mao Zedong era.
